# Proximalization of the distal anastomosis in frozen elephant trunk surgery

**DOI:** 10.1016/j.xjtc.2025.06.010

**Published:** 2025-06-25

**Authors:** Philipp Pfeiffer, Karen Wittemann, Edoardo Zancanaro, Vanessa Buchholz, Leon Mattern, Chris Probst, Franz Masseli, Ahmed Ghazy, Hendrik Treede, Daniel-Sebastian Dohle

**Affiliations:** Department of Cardiovascular Surgery, University Medical Center Mainz, Mainz, Germany

**Keywords:** aortic arch surgery, aortic dissection, thoracic aortic aneurysm, zone 0

## Abstract

**Background:**

The frozen elephant trunk (FET) technique is frequently used in aortic arch diseases. Proximalization of the distal anastomosis from zone 3 to zone 2 results in shorter distal ischemia times and improved outcomes. This study assessed the impact of performing distal anastomoses progressively more proximally.

**Methods:**

A total of 243 patients treated between March 2016 and December 2023 were identified using our institutional database, and relevant data were analyzed retrospectively. The patients were stratified according to the distal anastomosis zone, and perioperative and outcome variables were compared. A subgroup analysis was performed accordingly for patients with acute aortic dissections.

**Results:**

Most of the 243 included patients (66%) were treated for acute dissection, followed by chronic dissection (24%) and thoracic aortic aneurysms (10%). The patients’ mean age was 62.5 ± 10.8 years, and 175 patients (72%) were male. The distal ischemia time was significantly reduced with the more proximal anastomoses: 24.5 minutes for zone 0 (69 patients), 37.4 minutes for zone 1 (18 patients), 30.4 minutes for zone 2 (145 patients), and 38.7 minutes for zone 3 (11 patients); *P* < .001. A longer cross-clamping time in zone 0 was explained by the higher number of root procedures, while other outcome parameters showed no significant differences. These same significant differences also were present in the acute dissection subgroup. In a separate analysis, a shorter duration of distal ischemia correlated with improved long-term survival (*P* = .002).

**Conclusions:**

The FET technique with distal anastomosis in zone 2 is a reliable technique that produces good results. Proximalization of the distal anastomosis to zone 0 significantly reduces the ischemic burden and simplifies the procedure.

## Graphical Abstract

**Figure:**
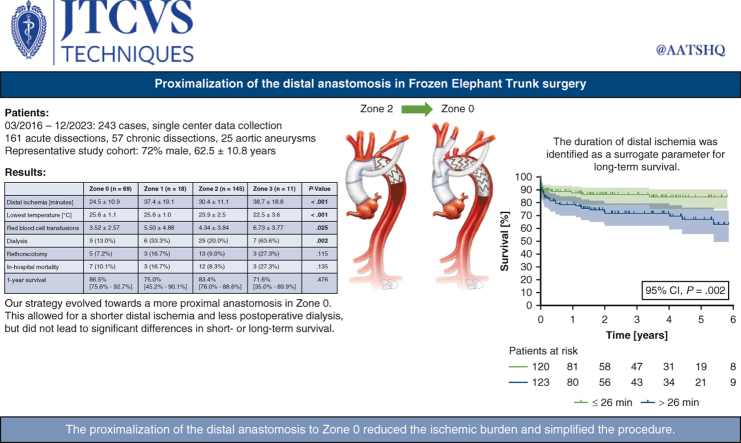

**Figure:**
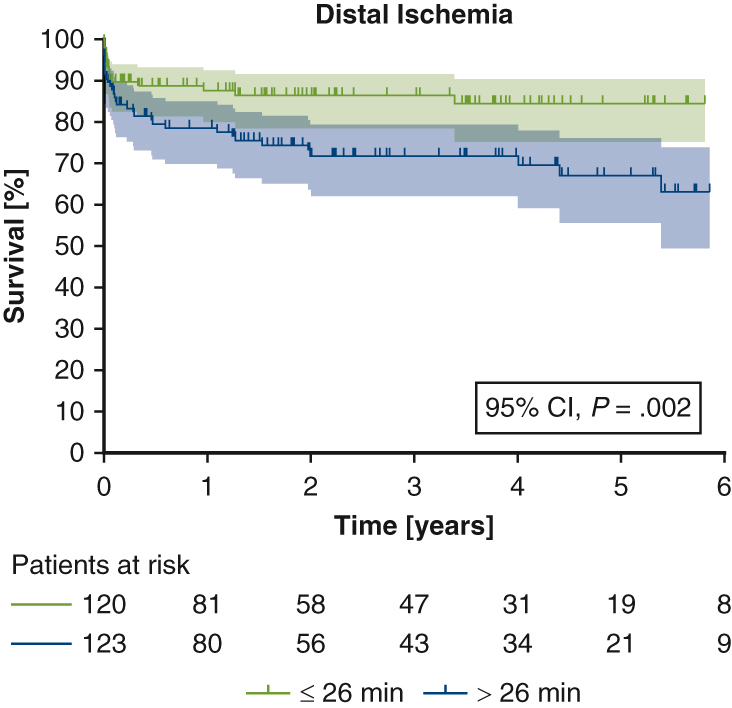
A shorter duration of distal ischemia significantly improved long-term survival.

**Central Message**

Proximalization of the distal anastomosis in frozen elephant trunk surgery to zone 0 reduced the ischemic burden, resulting in a shorter duration of distal ischemia.

**Perspective**

Frozen elephant trunk surgery is an established treatment option for aortic arch diseases. The procedure requires circulatory arrest, which inflicts a further ischemic burden on the patient in addition to the ischemic burden caused by the disease itself. This study analyzes whether proximalization of the distal anastomosis into zone 0 can reduce the ischemia and its sequelae.

The frozen elephant trunk (FET) prosthesis combines a traditional vascular prosthesis with a stent graft for treatment of aortic arch pathologies. Following its introduction in the early 2000s, FET has since been established as a treatment option for both dissections and aneurysms involving the aortic arch.^1^, ^2^, ^3^ The procedure constitutes major aortic surgery, as the distal anastomosis is generally performed in hypothermic circulatory arrest (HCA) with selective cerebral perfusion (SCP), and supra-aortic vessels are either replaced or reimplanted. The FET surgery, the underlying aortic disease itself, and cardiopulmonary bypass (CPB) impart a significant ischemic burden.

Different strategies to decrease the ischemic burden have been explored in contemporary research, most focusing on reducing the duration of HCA.^4^, ^5^, ^6^ Originally, the distal anastomosis was performed distal to the left subclavian artery (LSA) in zone 3 (Figure 1, *A*). One notable advancement was moving the distal anastomosis to zone 2 (Figure 1, *B*), along with different strategies for reinsertion or replacement of the supra-aortic vessels.^7^, ^8^, ^9^, ^10^ The more proximal location of the distal anastomosis, along with extraanatomic replacement of the LSA, allow for a quicker procedure, as the LSA can be anastomosed during reperfusion.^11^

**Figure 1:**
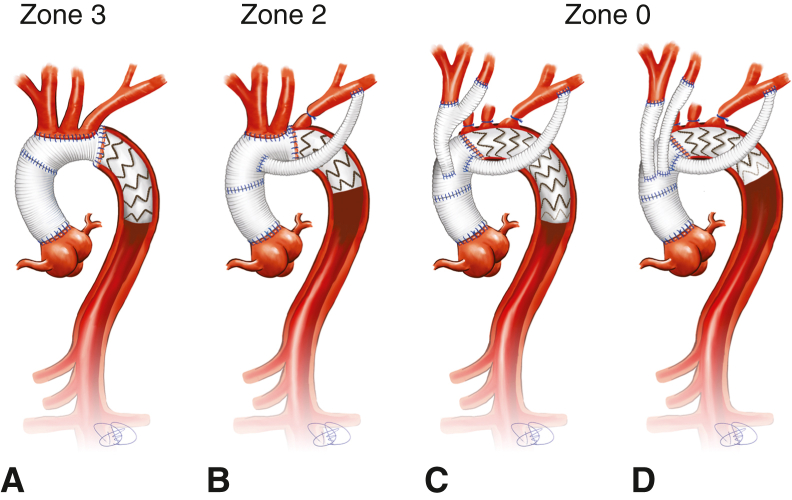
A, Zone 3, reimplantation of supra-aortic vessels using the island technique. B, Zone 2, reimplantation of innominate artery and left common carotid artery using the island technique, with extra-anatomic bypass of the left subclavian artery. C, Zone 0, innominate artery and left common carotid artery are supplied via branches of a trifurcate-style prosthesis, with extra-anatomic bypass of the left subclavian artery and a longer stent graft. D, Zone 0, innominate artery and left common carotid artery are separately replaced and implanted into a straight prosthesis, with extra-anatomic bypass of the left subclavian artery and a shorter stent graft.

Further proximalization into zone 0 (Figure 1, *C* and *D*), combined with debranching of the supra-aortic vessels during cooling, potentially can provide further reduction of distal ischemia and CPB time, as the more proximal location of the distal anastomosis decreases the difficulty of the procedure. However, the more rigid stent graft covers a greater portion of the angled aortic arch, possibly influencing aortic remodeling.^12^^,^^13^

This approach has been investigated by different groups with promising results, although some studies are limited by their number of patients and the duration of follow-up.^14^, ^15^, ^16^ A further analysis of whether the zone 0 approach translates to an improved outcome is warranted.

Several configurations of FET prostheses are commercially available to cater to different strategies of aortic arch replacement and supra-aortic vessel treatment. The zone of the distal anastomosis, the configuration of the implanted FET graft, and the strategy of supra-aortic vessel treatment are important factors in FET surgery. The present retrospective study aimed to assess the impact of performing the distal anastomosis progressively more proximally on the surgical procedure, postoperative course, and outcome.

## Materials and Methods

### Ethical Review

This study was approved by the Institutional Review Board of the Medical Association of the state of Rheinland-Pfalz, Germany (2018-13574-Epidemiologie) on August 9, 2018, and was performed in accordance with the Declaration of Helsinki. Owing to the retrospective data collection, the need for informed patient consent was waived.

### Patients

Patients undergoing thoracic aortic surgery are prospectively documented in our institutional aortic surgery database. All patients who underwent FET surgery for any indication between March 2016 and December 2023 were included in the study. The relevant data, consisting of demographics, comorbidities, procedural details, postoperative complications, follow-up, and survival, were retrieved from the database for retrospective analysis.

The primary endpoints of the study were short- and long-term outcomes compared between the 4 different zones for the distal anastomosis. Additionally, a descriptive analysis of further perioperative details, including possible confounding variables, is provided.

### Surgical Strategy

All procedures were performed at a single center by cardiac surgeons. Although some details varied over the course of almost 8 years and among individual surgeons and patients, the FET procedures at our center followed a common standard procedure, as follows. Preoperative imaging included computed tomography angiography, and aortic arch replacement was indicated owing to extension of the aneurysm or dissection into the aortic arch. Three arterial lines (both radial arteries and the left femoral artery), a central venous line, and a high-volume intravenous access (eg, Shaldon catheter) were placed routinely. Intraoperative monitoring included cerebral oximetry using bilateral near-infrared spectroscopy (Medtronic), vesical core body temperature measurements, and transesophageal echocardiography.

If suitable, the right subclavian artery was used for arterial cannulation prior to sternotomy. After the initiation of CPB, the patient was cooled to the desired temperature. During this period before cardiac arrest, debranching of the supra-aortic vessels was begun, typically starting with the left carotid and subclavian arteries. After aortic cross-clamping, the aortic root replacement was prepared if necessary. Once the target temperature was reached, the cross-clamp was released, and the brachiocephalic trunk was clamped, thereby starting both SCP and distal ischemia. Near-infrared spectroscopy was used to verify adequate oxygenation of both hemispheres, and bilateral cerebral perfusion was established if significant differences were observed. The arch was resected to the predetermined zone, and the selected FET prosthesis was implanted in high-moderate HCA, typically aiming for 24 to 26 °C.

During completion of the distal anastomosis, a Foley catheter was placed antegrade into the distal stent graft, expanded, and connected to an additional arterial line to restart distal perfusion. The supra-aortic vessels were either reimplanted or anastomosed with the respective branches of the FET prosthesis. The Foley catheter was then removed, and after deairing, the prosthesis was cannulated and clamped to begin whole body perfusion and rewarming. During rewarming, the proximal repair was completed, and the LSA was bypassed extra-anatomically. After CPB weaning and ensuring hemostasis, the sternal wound was closed, and the patient was transferred to the intensive care unit for further monitoring and treatment.

### Statistical Analysis

The patients were stratified according to the site of distal anastomosis into 4 groups (zones 0, 1, 2, and 3) for comparison. The same comparisons were also conducted for the subgroup of patients with acute aortic dissection (AAD). Because the zone 0 and zone 2 groups composed the majority of the study cohort (88%), a direct comparison of these 2 groups was conducted in addition to the 4-group comparison. The statistical analysis was performed using SPSS version 29.0.2.0 (IBM), Prism version 10.0.0 (GraphPad Software), and Wizard Pro version 1.9.7 (Evan Miller).

For the 4-group analysis, the normal distribution of continuous variables was assessed using the Shapiro-Wilk test, and the homogeneity of variance was assessed using the Levene test. The groups were compared using analysis of variance if both conditions were met and using the Kruskal-Wallis test if either condition was not met. The Student *t* test was used to compare continuous variables between 2 groups. The χ^2^ test and Fisher exact test were used to compare frequency variables as appropriate. Survival was compared using the log-rank test for categorial variables and the Cox proportional hazards model for metric variables. The accompanying Kaplan-Meier curves were created using GraphPad Prism and were truncated when fewer than 10 patients at risk remained.

All tests were 2-sided with a 95% confidence interval (CI), and the α level was set at 0.05 for statistical significance. Frequency data are presented as absolute number (percentage), continuous data are presented as mean ± standard deviation, or if the mean was not a representative description due to outliers, as median (interquartile range), and survival rates are presented as percentage (95% CI). Statistically significant *P* values are in bold type.

## Results

### Demographics and Comorbidities

Between March 2016 and December 2023, 243 consecutive patients were treated surgically using the FET technique. Patient demographics and comorbidities of the entire study cohort are presented in Table 1. The distribution of AADs and chronic aortic dissections (CADs), as well as thoracic aortic aneurysms, was similar across the 4 different zones of the distal anastomoses. Patients in the zone 0 and zone 2 groups were generally younger (*P* = .030), while the sex distribution was similar across the 4 groups, with an overall higher number of males than females. The body surface area was higher in patients with distal anastomosis in zone 0 and zone 2 compared to those with distal anastomosis in zones 1 and 3 (*P* = .042), while the body mass index showed a similar trend without reaching statistical significance (*P* = .051). Hypertension was the most common comorbidity in all 4 groups, and the prevalence of other cardiovascular comorbidities was distributed evenly among the 4 groups. The rate of reoperation corresponded to the prevalence of CAD.

**Table 1:** Demographics

Parameter	Total (N = 243)	Zone 0 (N = 69)	Zone 1 (N = 18)	Zone 2 (N = 145)	Zone 3 (N = 11)	*P* value
Disease, n (%)						.279
AAD	161 (66.3)	45 (65.2)	11 (61.1)	96 (66.2)	9 (81.8)	
CAD	57 (23.5)	21 (30.4)	5 (27.8)	30 (20.7)	1 (9.1)	
TAA	25 (10.3)	3 (4.3)	2 (11.1)	19 (13.1)	1 (9.1)	
Age, y, mean ± SD	62.5 ± 10.8	61.6 ± 11.1	66.2 ± 10.9	61.8 ± 10.3	71.4 ± 10.2	**.030**
Sex, n (%)						.869
Male	175 (72.0)	48 (69.6)	13 (72.2)	105 (72.4)	9 (81.8)	
Female	68 (28.0)	21 (30.4)	5 (27.8)	40 (27.6)	2 (18.2)	
BMI, kg/m^2^, mean ± SD	27.7 ± 5.4	28.3 ± 5.6	25.7 ± 4.9	27.9 ± 5.5	25.3 ± 2.7	.051
BSA, m^2^, mean ± SD	2.04 ± 0.26	2.08 ± 0.24	1.90 ± 0.26	2.05 ± 0.26	1.95 ± 0.18	**.042**
Medical history, n (%)						
Hypertension	191 (78.6)	53 (76.8)	14 (77.8)	116 (80.0)	8 (72.7)	.852
Diabetes mellitus	14 (5.8)	6 (8.7)	0 (0.0)	6 (4.1)	2 (18.2)	.091
Nicotine	64 (26.3)	21 (30.4)	6 (33.3)	37 (25.5)	0 (0.0)	.136
Coronary artery disease	36 (14.8)	12 (17.14)	4 (22.2)	19 (13.1)	1 (9.1)	.598
COPD	18 (7.4)	3 (4.3)	1 (5.6)	14 (9.7)	0 (0.0)	.537
Renal Insufficiency	23 (9.5)	6 (8.7)	2 (11.1)	15 (10.3)	0 (0.0)	.834
Previous aortic or cardiac surgery	79 (32.5)	22 (31.9)	5 (27.8)	50 (34.5)	2 (18.2)	.753

Bold type indicates statistical significance. *AAD*, Acute aortic dissection; *CAD*, chronic aortic dissection; *TAA*, thoracic aortic aneurysm; *BMI*, body mass index; *BSA*, body surface area; *COPD*, chronic obstructive pulmonary disease.

In the subgroup analysis of AAD patients, the demographic characteristics were distributed homogenously across the 4 different groups (Table E1). The entire group of AAD patients can be characterized adequately using the Penn classification, which showed a typical distribution. Approximately 38% of AAD patients were classified as Penn A (without shock and malperfusion), while 37% had localized malperfusion (Penn B), 8% were in shock (Penn C), and 17% had both shock and localized malperfusion (Penn BC).

### Operative Details

The operative details of the entire cohort are summarized in Table 2, and the subgroup analysis of AAD patients is presented in Table E2. During the study period, we observed a proximalization of the distal anastomosis from zone 3 to zone 0 (2016: 100% zone 3; 2018: 98% zone 2; 2023: 79% zone 0). Corresponding with the more frequent use of the longer E-vita open neo trifurcate in zone 0, the mean stent length was increased significantly (*P* = .001). The diameters of the vascular and stent graft portions of the FET were consistent across all zones at 28 mm (*P* = .193) and 29 mm (*P* = .113), respectively. Although the CPB time did not differ significantly among the groups (*P* = .550), a significant increase in cardiac ischemia time with longer cross-clamping time was observed with proximalization, particularly when comparing zone 0 to zone 2 (154 ± 49 minutes vs 137 ± 43 minutes; *P* = .029). This correlates with a higher number of concomitant root procedures (Bentall and David operations, as well as isolated noncoronary sinus replacement) in zone 0 (*P* = .002).

**Table 2:** Operative details

Parameter	Total (N = 243)	Zone 0 (N = 69)	Zone 1 (N = 18)	Zone 2 (N = 145)	Zone 3 (N = 11)	*P* value
FET vascular graft diameter, mm, mean ± SD	28.4 ± 3.1	28.4 ± 1.5	29.2 ± 1.5	28.2 ± 3.6	28.2 ± 5.4	.193
FET stent graft diameter, mm, mean ± SD	28.6 ± 3.5	28.9 ± 3.4	29.8 ± 2.5	28.4 ± 3.6	28.6 ± 5.3	.113
FET stent graft length, mm, mean ± SD	138.3 ± 22.6	153.6 ± 29.4	152.5 ± 28.6	130.4 ± 11.2	124.6 ± 12.1	**.001**
FET device, n (%)						**<.001**
E-vita open neo	78 (32.1)	62 (89.9)	11 (61.1)	5 (3.4)	0 (0.0)	
E-vita open	154 (63.4)	6 (8.7)	6 (33.3)	133 (91.7)	9 (81.8)	
Thoraflex	11 (4.5)	1 (1.4)	1 (5.6)	7 (4.8)	2 (18.2)	
FET configuration, n (%)						**<.001**
Straight	181 (74.5)	30 (43.5)	8 (44.4)	134 (92.4)	9 (81.8)	
Trifurcated	51 (21.0)	38 (55.1)	9 (50.0)	4 (2.8)	0 (0.0)	
Branched	11 (4.5)	1 (1.4)	1 (5.6)	7 (4.8)	2 (18.2)	
Operative details, mean ± SD						
CPB time, min	263.0 ± 60.4	257.1 ± 54.0	279.3 ± 61.0	263.2 ± 63.4	269.6 ± 59.6	.550
Cross-clamp time, min	144.1 ± 46.3	153.5 ± 49.1	164.9 ± 54.9	136.9 ± 43.0	145.7 ± 39.4	**.029**
Distal ischemia time, min	29.6 ± 12.7	24.5 ± 10.9	37.4 ± 19.1	30.4 ± 11.1	38.7 ± 18.8	**<.001**
SCP time, min	57.6 ± 20.6	57.4 ± 19.9	66.4 ± 27.7	55.4 ± 18.7	74.7 ± 26.7	**.050**
Lowest temperature, °C	24.5 ± 2.4	25.6 ± 1.1	25.6 ± 1.0	23.9 ± 2.5	22.5 ± 3.6	**<.001**
Transfusions and coagulation factors, mean ± SD						
pRBC, 250 mL units	4.30 ± 3.66	3.52 ± 2.57	5.50 ± 4.88	4.34 ± 3.84	6.73 ± 3.77	**.025**
Platelets, 250 mL units	2.02 ± 1.152	1.87 ± 1.06	2.22 ± 1.17	2.01 ± 1.17	2.73 ± 1.27	.160
FFP, 250 mL units	3.07 ± 3.09	3.62 ± 3.12	3.50 ± 2.50	2.83 ± 3.13	2.00 ± 2.86	.064
Fibrinogen, g	4.55 ± 2.40	4.36 ± 2.50	5.06 ± 2.24	4.54 ± 2.36	5.09 ± 2.70	.898
PCC, IU	2679 ± 1728	2642 ± 1715	2589 ± 1661	2652 ± 1759	3418 ± 1543	.488
Arterial cannulation site, n (%)						**.038**
Subclavian/axillary	205 (84.4)	57 (82.6)	14 (77.8)	127 (87.6)	7 (63.6)	
DTLC	28 (11.5)	8 (11.6)	2 (11.1)	16 (11.0)	2 (18.2)	
Others	10 (4.1)	4 (5.8)	2 (11.1)	2 (1.4)	2 (18.2)	
Aortic valve intervention, n (%)						**.023**
Replacement	40 (16.5)	16 (23.2)	4 (22.2)	20 (13.8)	0 (0.0)	
Reconstruction	108 (44.4)	21 (30.4)	5 (27.8)	76 (52.4)	6 (54.5)	
Aortic root intervention, n (%)						**.002**
Bentall	29 (11.9)	12 (17.4)	2 (11.1)	15 (10.3)	0 (0.0)	
David	11 (4.5)	6 (8.7)	1 (5.6)	4 (2.8)	0 (0.0)	
Isolated sinus replacement, n (%)	20 (8.2)	13 (18.8)	0 (0.0)	7 (4.8)	0 (0.0)	
Concomitant TEVAR, n (%)	14 (5.8)	3 (4.3)	2 (11.1)	9 (6.2)	0 (0.0)	.573

Bold type indicates statistical significance. *FET*, Frozen elephant trunk; *CPB*, cardiopulmonary bypass; *SCP*, selective cerebral perfusion; *pRBC*, packed red blood cells; *FFP*, fresh frozen plasma; *PCC*, prothrombin complex concentrate; *DTLC*, direct true lumen cannulation; *TEVAR*, thoracic endovascular aortic repair.

The duration of SCP was significantly longer in zone 3 compared to zones 0 and 2 (*P* = .050). Proximalization of the distal anastomosis resulted in a significant reduction of the distal ischemia time from 39 minutes in zone 3 to 25 minutes in zone 0 (*P* < .001), with 23% of cases in zone 0 taking no longer than 15 minutes. Concurrently, the lowest measured core body temperature during HCA increased significantly, from 23 °C to 26 °C (*P* < .001). This change from low-moderate to high-moderate hypothermia was enabled by the decreased distal ischemia durations as the operation progressed more swiftly. The slightly longer durations of CPB, distal ischemia, and SCP in zone 1 compared to zone 2 did not reach statistical significance in direct comparisons. The amount of intraoperative transfusions of packed red blood cells (pRBC) was lowest in zone 0 (*P* = .025). A similar trend was observed for platelets and fibrinogen, but statistical significance was not reached.

The AAD subgroup demonstrated a similar distribution of data, with comparable CPB times across all groups (*P* = .186) and longer cross-clamping times (*P* = .001) and shorter distal ischemia times in the zone 0 group (*P* < .001). The shorter distal ischemia time allowed for a higher temperature in this subgroup (*P* < .001). Likewise, the longer cross-clamping times in zone 0 were accompanied by a higher proportion of concomitant aortic root procedures, exceeding 50% in zone 0 (*P* = .001). While the trend toward lower transfusions (pRBC, platelets) was also observed in the AAD subgroup, the differences were not statistically significant. Conversely, the number of fresh frozen plasma units transfused was significantly lower in the zone 3 group (*P* = .024), which likely can be attributed to the low number of patients in this group. The number of fresh frozen plasma units transfused did not differ significantly among the zone 0, zone 1, and zone 2 groups.

In both the entire study cohort and the AAD subgroup, 6% of patients received a distal extension using thoracic endovascular aortic repair (TEVAR) during the same hospital stay, with no significant differences by zone of distal anastomosis.

### Outcomes

**Figure 2:**
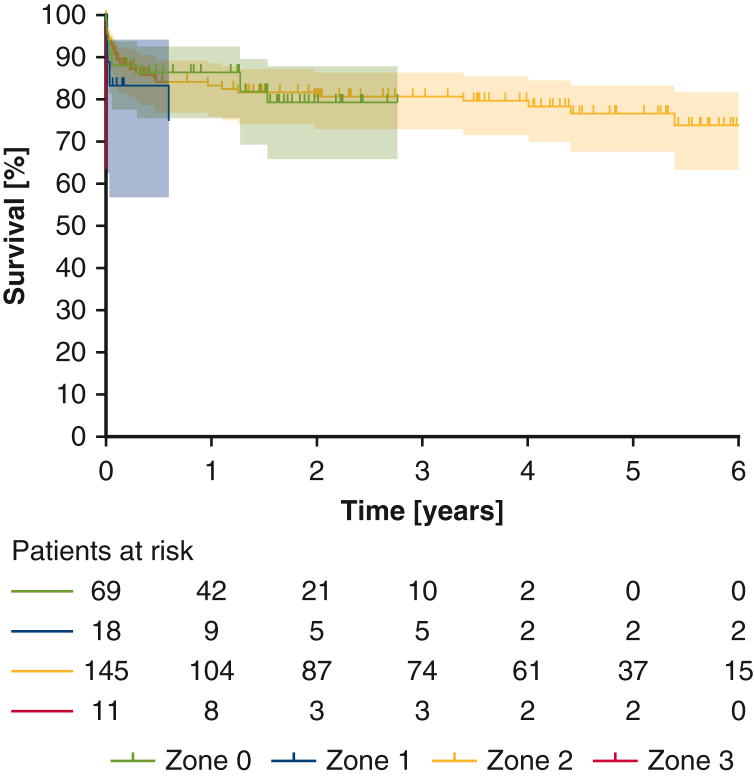
Long-term survival stratified by the zone of distal anastomosis. Shaded area indicates 95% confidence interval.

There were no significant differences among the 4 groups in the major outcome variables (Table 3), including in-hospital mortality (zone 0, 10%; zone 1, 17%; zone 2, 8%; zone 3, 27%; *P* = .135), new neurologic deficits (zone 0, 9%; zone 1, 17%; zone 2, 9%; zone 3, 0%; *P* = .545), and repeat thoracotomy rate (zone 0, 10%; zone 1, 17%; zone 2, 9%; zone 3, 27%; *P* = .115). Neurologic deficits were mostly caused by perioperative stroke, with a lower occurrence of spinal cord ischemia or peripheral nerve damage. Similarly, during follow-up (median follow-up, 2.43 years; interquartile range, 1.76-3.11 years), no significant survival differences were found (1-year survival, 83.1% [95% CI, 77.6%-87.4%]; 3-year survival, 78.9% [95% CI, 72.8%-83.9%]; *P* = .438) (Figure 2). However, a significantly reduced rate of postoperative renal failure necessitating new-onset dialysis was observed in the zone 0 group compared to the zone 3 group (*P* = .002). The remainder of the postoperative course, including postoperative mechanical ventilation and intensive care unit and hospital length of stay, revealed no significant differences, neither in the overall cohort nor in the subgroup analysis of AAD (Table E3). As we shifted the distal anastomosis toward zone 0, the follow-up in these more recently operated patients was less extensive.

**Table 3:** Outcomes

Parameter	Total (N = 243)	Zone 0 (N = 69)	Zone 1 (N = 18)	Zone 2 (N = 145)	Zone 3 (N = 11)	*P* value
Postoperative ventilation, h, median (IQR)	24.0 (14.0-86.0)	22.0 (12.5-45.5)	21.5 (14.3-139.3)	23.5 (15.0-69.5)	54.0 (24.8-113.5)	.252
ICU stay, d, mean ± SD	6.7 ± 6.1	6.5 ± 6.1	7.5 ± 6.9	6.6 ± 6.0	7.6 ± 6.9	.820
Hospital stay, d, mean ± SD	16.8 ± 11.8	15.0 ± 10.5	18.4 ± 11.2	16.7 ± 9.3	26.7 ± 32.0	.182
Rethoracotomy, n (%)	24 (9.9)	5 (7.2)	3 (16.7)	13 (9.0)	3 (27.3)	.115
In-hospital mortality, n (%)	25 (10.3)	7 (10.1)	3 (16.7)	12 (8.3)	3 (27.3)	.135
Tracheotomy, n (%)	29 (11.9)	7 (10.1)	3 (16.7)	16 (11.0)	3 (27.3)	.296
Distal reintervention (TEVAR), n (%)	43 (17.7)	12 (17.4)	5 (27.8)	24 (16.6)	2 (18.3)	.655
Dialysis, n (%)	51 (21.0)	9 (13.0)	6 (33.3)	29 (20.0)	7 (63.6)	**.002**
New neurologic deficit, n (%)	22 (9.1)	6 (8.7)	3 (16.7)	13 (9.0)	0 (0.0)	.545
Stroke, n (%)∗	18 (81.8)	5 (83.3)	3 (100.0)	10 (76.9)	0 (0.0)	1.000
Spinal cord injury, n (%)∗	5 (22.7)	0 (0.0)	1 (33.3)	4 (30.8)	0 (0.0)	.345
Long-term survival, % (95% CI)						.438
1 y	83.1 (77.6-87.4)	86.5 (75.6-92.7)	75.0 (45.2-90.1)	83.4 (76.0-88.6)	71.6 (35.0-89.9)	
2 y	78.9 (72.8-83.9)	79.4 (65.9-88.0)	75.0 (45.2-90.1)	80.8 (73.0-86.5)	47.7 (8.6-80.0)	
3 y	78.9 (72.8-83.9)	79.4 (65.9-88.0)	75.0 (45.2-90.1)	80.8 (73.0-86.5)	47.7 (8.6-80.0)	
4 y	78.0 (71.6-83.1)	79.4 (65.9-88.0)	75.0 (45.2-90.1)	79.7 (71.6-85.7)	47.7 (8.6-80.0)	
5 y	75.2 (67.7-81.2)	N/A	75.0 (45.2-90.1)	76.7 (67.7-83.5)	47.7 (8.6-80.0)	
6 y	72.7 (63.6-79.9)	N/A	75.0 (45.2-90.1)	73.9 (63.4-81.9)	N/A	

Bold type indicates statistical significance. *IQR*, Interquartile range; *ICU*, intensive care unit; *TEVAR*, thoracic endovascular aortic repair; *N/A*, not applicable.

∗ Only including patients with new neurologic deficits (n = 22).

A total of 43 patients (18%) required a distal reintervention by TEVAR extension during the follow-up period, including those treated during the initial hospital stay. Of these, 11 (26%) were initially planned to undergo a 2-stage procedure. A distal stent-induced new entry (dSINE) was the sole indication for 23% (n = 10) of the distal reinterventions. Among AAD patients, the distal reintervention rate was 12% (n = 19), with dSINE present in 26% (n = 5) of the cases. There were no significant differences in TEVAR extension rate among the different groups.

### Zone 0 Versus Zone 2

A direct comparison of the zone 0 and zone 2 groups (Table E4) confirmed the reduced distal ischemia (*P* < .001) and the switch from low moderate to high moderate HCA (*P* < .001) for zone 0. As in the total study cohort, the significantly higher number of root procedures (*P* < .001) was accompanied by a longer duration of CPB (*P* = .013). The short- and long-term outcomes revealed no significant differences between the zone 0 and zone 2 groups.

## Discussion

Reducing the ischemic burden has been an ongoing concern in FET surgery, with various groups exploring different techniques and strategies. A pivotal shift involved proximalization of the distal anastomosis from zone 3 to zone 2. In 2015, Tsagakis and colleagues^7^ demonstrated reduction of the distal ischemia close to 30 minutes by proximalization of the distal anastomosis to zone 2 and extra-anatomic rerouting of the LSA. In a cohort of 92 patients, Detter and colleagues^17^ demonstrated that this adjustment cut the distal ischemia time from 77 minutes to 42 minutes. In a larger cohort of 357 patients, a reduction in ischemia time from 77 minutes to 32 minutes and an increase in core temperature from 25 °C to 28 °C were achieved by the Essen group.^18^ The introduction of a 4-site perfusion method significantly improved outcomes, enhancing a combined clinical endpoint of 90-day mortality, disabling strokes, and postoperative complications.

Alternative methods include hybrid techniques to save ischemia time during supra-aortic vessel interventions. Pichlmaier and colleagues^19^ replaced traditional anastomoses with Viabahn stents (WL Gore) in a series of 112 patients, which maintained distal ischemia at 60 minutes with 71 minutes of selective antegrade cerebral perfusion. Innovations continued with the integration of up to 2 inner branches for the left carotid and subclavian arteries, culminating in a distal anastomosis in zone 0, which decreased ischemia time to 38 minutes.^20^

A French team developed a technique to start reperfusion earlier by achieving hemostasis by encircling the arch with a tourniquet in zones 1 and 2, allowing distal anastomosis during antegrade reperfusion.^6^ This method reduced ischemia to 5 minutes at a core body temperature of 34 °C.

Recent developments from 2 Italian groups have further innovated the field by introducing a technique that involves retrograde perfusion via a femoral artery and endoclamping of an endograft in the arch.^4^^,^^5^ This approach completely eliminates distal ischemia during debranching and replacement, although limitations in cases of dissection have been noted by Berretta and colleagues.^4^

With the introduction of the E-vita open neo (Artivion) in 2020, we significantly altered our surgical strategy. Previously our approach primarily involved reimplanting the head and neck vessels as an island of the innominate artery and the left carotid artery with distal anastomosis in zone 2.^8^ We transitioned to a new strategy that involves debranching the supraaortic vessels and performing distal anastomosis in zone 0. To save cardiac ischemia time, we performed the debranching procedure prior to the onset of cardiac ischemia whenever possible. The debranching-first technique has been established as a safe and feasible method in smaller trials and case reports.^21^, ^22^, ^23^ We aimed to cool the patient as quickly as possible during the debranching, which allowed us to proceed with the arch procedure in HCA shortly after cross-clamping. This procedure was facilitated by the early placement of a Foley catheter and prompt resumption of distal perfusion, which kept distal ischemia times short. Overall, this approach allowed for an early start of the rewarming process, thereby effectively shortening the CPB times.

Interestingly, this reduction in ischemia is reflected only in the distal ischemia times. Paradoxically, cross-clamp times were significantly longer in the zone 0 group. However, at the same time the rate of root procedures increased, with root procedures performed in >50% of AAD patients in the zone 0 group but in <20% of those in the zone 2 group.

To eliminate the effect of root procedures, we analyzed patients with and without root procedures separately (Table 4). These isolated analyses showed no significant differences in the duration of CPB and cross-clamp times, whereas distal ischemia time remained significantly shorter in the zone 0 group in both patients with root procedures (*P* = .002) and those without root procedures (*P* = .001). Therefore, the longer cross-clamp times in zone 0 can be attributed mainly to the higher rate of root procedures.

**Table 4:** Operative duration in patients with and without root procedure

Parameter	Total	Zone 0	Zone 1	Zone 2	Zone 3	*P* value
Patients with root procedure	N = 60	N = 31	N = 3	N = 26	N = 0	
CPB time, min, mean ± SD	275 ± 65	268 ± 57	278 ± 50	284 ± 76		.839
Cross-clamp time, min, mean ± SD	178 ± 44	173 ± 40	212 ± 34	179 ± 48		.335
Distal ischemia time, min, mean ± SD	26 ± 12	22 ± 12	19 ± 4	31 ± 11		**.002**
Patients without root procedure	N = 183	N = 38	N = 15	N = 119	N = 11	
CPB time, min, mean ± SD	259 ± 58	249 ± 51	280 ± 64	259 ± 60	270 ± 60	.343
Cross-clamp time, min, mean ± SD	133 ± 42	137 ± 50	155 ± 54	128 ± 36	146 ± 39	.160
Distal ischemia time, min, mean ± SD	31 ± 13	26 ± 10	41 ± 19	30 ± 11	39 ± 19	**.001**

Bold type indicates statistical significance. *CPB*, Cardiopulmonary bypass; *SD*, standard deviation.

Furthermore, the modest increase in cross-clamp time of 17 minutes is insufficient to accommodate an additional root procedure in every second patient, and the CPB times remained nearly identical.^24^ In summary, we interpret these findings as a positive effect of the optimized workflow of the zone 0 strategy: the simpler distal anastomosis in zone 0 reduced the duration of distal ischemia. Earlier rewarming reduced the need for prolonged reperfusion at the end of the operation to achieve normothermia. By reducing cardiac ischemia time during the arch procedure at the beginning of the operation, the total duration of cardiac ischemia and CPB can be better estimated, allowing for the safe performance of the FET operation with concomitant root procedures as necessary, especially in AAD patients. The lower pRBC transfusions in the zone 0 group also indicate a reduction of ischemic burden.^25^

Notably, these proximalization effects are observable between zone 0 and other zones but not for the proximalization from zone 2 to zone 1. This likely can be attributed to the smaller number of patients in the zone 1 group, their older age, and, in AAD patients, their higher rates of localized and generalized malperfusion as assessed by the Penn classification.

The short- and long-term survival of the zone 0 group and of the entire study cohort are comparable to previously reported outcomes of larger register studies.^26^ Although only secondary endpoints, such as the duration of distal ischemia and the rate of postoperative kidney failure necessitating new-onset dialysis, have shown statistically significant improvements, we deduce that the combination of strategies that we described with distal anastomosis in zone 0 can reduce the ischemic burden.

**Figure 3:**
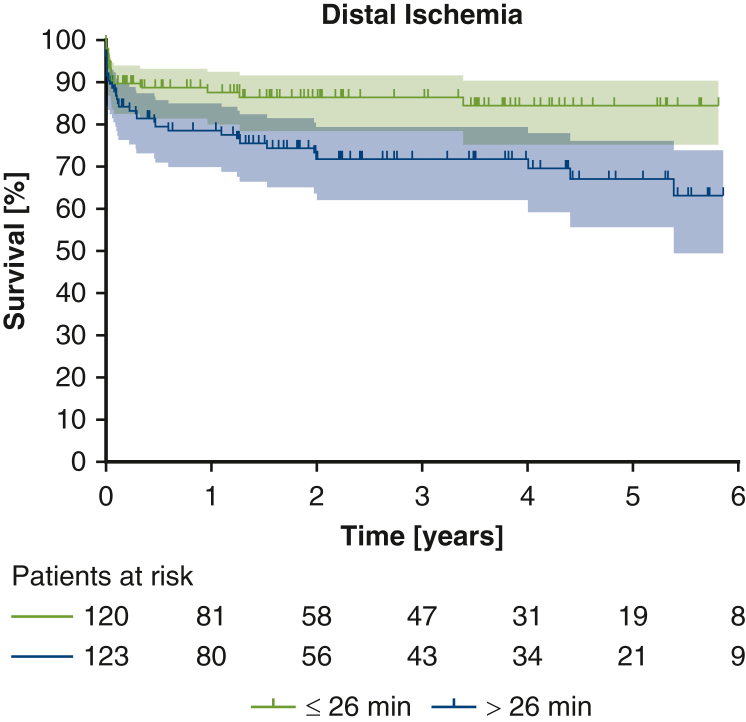
Long-term survival stratified by the median duration of distal ischemia. Shaded area indicates 95% confidence interval.

To assess the influence of the ischemic burden caused by the surgery, as quantified by the duration of distal ischemia, on long-term survival, we used a Cox proportional hazards model to relate these 2 variables. This analysis revealed significantly longer survival in patients with a shorter distal ischemia time (*P* = .002; Figure 3), indicating that the duration of distal ischemia might constitute a predictive factor.

Although our study likely lacked the power to demonstrate a direct survival benefit for zone 0 patients, the significantly shorter distal ischemia may be considered as a surrogate parameter and potentially could translate to improved long-term survival in a larger study cohort. The heterogenous study collective containing patients with AAD and CAD, as well as thoracic aortic aneurysms, might require a larger number of patients or a longer duration of follow-up, to show significant survival benefits. Furthermore, circulatory shock and localized malperfusion in AAD constitute confounding variables, as they have a proven influence on mortality.^27^, ^28^, ^29^, ^30^ Nevertheless, the fact that distal ischemia and dialysis rates were significantly lower in both the overall cohort and the AAD subgroup gives credence to our hypothesis of a reduced ischemic burden with the more proximal anastomosis.

Moreover, the technique proposed here with proximate anastomoses is easily reproducible even by inexperienced aortic surgeons. Concerns about increased reintervention rates because of potentially higher tension at the distal end of the stent with distal anastomosis in zone 0 were not confirmed in this work, as similar reintervention rates were observed. Similarly, the greater amount of prosthetic material due to debranching of the supra-aortic vessels did not result in higher rates of strokes or other complications.

In summary, we maintain that the technique presented here, which involves even earlier distal reperfusion, can reduce the ischemic burden in FET surgery and is readily adaptable without compromising safety and outcomes.

### Limitations

The relevance of our present findings is limited by the retrospective data collection. The comparatively low number of patients in the zone 3 group and, to a lesser extent, the zone 1 group make these groups susceptible to outliers. Additionally, the high number of patients with AAD, who often present with impaired circulation, may introduce a confounding factor, as their predisposition to end-organ ischemia could influence outcomes independent of the surgery. Other abdominal organs and laboratory values were not assessed in this study. The FET graft and zone of distal anastomosis were selected at the discretion of the surgeon, and thus a selection bias cannot be ruled out. The duration of follow-up is restricted due to the relatively recent study period. The longer stent graft in zone 0 might have influenced aortic remodeling, but computed tomography scans were not analyzed for this study.^31^ Therefore, further studies are needed to confirm our observations and evaluate possible differences regarding long-term survival, reinterventions, and aortic remodeling in a larger study population.

## Conclusions

The FET procedure constitutes major aortic surgery, and the suitable zone for the proximal anastomosis must be selected after careful consideration, taking into account the anticipated extent of aortic repair and the treatment of supra-aortic vessels. Zone 2 remains a reliable technique with good results. The distal anastomosis can be safely moved more proximally into zone 0 without influencing stroke or mortality rates. The easier anatomy enables a quicker procedure, thus reducing the ischemic burden, as evidenced by decreased postoperative renal failure requiring dialysis. If necessary, aortic root replacement can be performed without a risky increase in the duration of CPB.

## Conflict of Interest Statement

Dr Dohle served as a consultant to Artivion during the study period. All other authors reported no conflicts of interest.

The *Journal* policy requires editors and reviewers to disclose conflicts of interest and to decline handling or reviewing manuscripts for which they may have a conflict of interest. The editors and reviewers of this article have no conflicts of interest.

## Figures and Tables

**Table E1:** Demographics and preoperative status, AAD subgroup

Parameter	Total AAD (N = 161)	Zone 0 (N = 45)	Zone 1 (N = 11)	Zone 2 (N = 96)	Zone 3 (N = 9)	*P* value
Age, y, mean ± SD	62.1 ± 10.5	62.9 ± 10.1	65.9 ± 10.8	60.6 ± 10.5	69.4 ± 10.0	.093
Sex, n (%)						.892
Male	121 (75.2)	32 (71.1)	8 (72.7)	74 (77.1)	7 (77.8)	
Female	40 (24.8)	13 (28.9)	3 (27.3)	22 (22.9)	2 (22.2)	
BMI, kg/m^2^, mean ± SD	28.0 ± 5.5	28.5 ± 6.0	26.7 ± 4.9	28.2 ± 5.5	25.9 ± 2.6	.319
BSA, m^2^, mean ± SD	2.06 ± 0.25	2.08 ± 0.25	1.95 ± 0.27	2.07 ± 0.25	1.98 ± 0.19	.319
Medical history, n (%)						
Hypertension	121 (75.2)	33 (73.3)	7 (63.6)	75 (78.1)	6 (66.7)	.571
Diabetes mellitus	10 (6.2)	5 (11.1)	0 (0.0)	4 (4.2)	1 (11.1)	.260
Nicotine	39 (24.2)	12 (26.7)	4 (36.4)	23 (24.0)	0 (0.0)	.262
Coronary artery disease	18 (11.2)	8 (17.8)	1 (9.1)	8 (8.3)	1 (11.1)	.338
COPD	15 (9.3)	3 (6.7)	1 (9.1)	11 (11.5)	0 (0.0)	.737
Renal insufficiency	13 (8.1)	1 (2.2)	1 (9.1)	11 (11.5)	0 (0.0)	.217
Previous aortic or cardiac surgery	12 (7.5)	3 (6.7)	0 (0.0)	8 (8.3)	1 (11.1)	.812
Penn classification, n (%)						.259
A	61 (37.9)	20 (44.4)	2 (18.2)	35 (36.5)	4 (44.4)	
B	59 (36.6)	18 (40.0)	4 (36.4)	36 (37.5)	1 (11.1)	
C	13 (8.1)	2 (4.4)	2 (18.2)	7 (7.3)	2 (22.2)	
BC	28 (17.4)	5 (11.1)	3 (27.3)	18 (18.8)	2 (22.2)	
CPR, n (%)	16 (9.9)	3 (6.7)	0 (0.0)	9 (9.4)	4 (44.4)	**.019**
Aortic regurgitation (≥II°), n (%)	84 (52.5)	21 (46.7)	2 (18.2)	59 (61.5)	2 (22.2)	**.006**
True lumen collapse, n (%)	72 (44.7)	21 (46.7)	4 (36.4)	44 (45.8)	3 (33.3)	.850
Malperfusion, n (%)	87 (54.0)	23 (51.1)	7 (63.6)	54 (56.3)	3 (33.3)	.522
Coronary	25 (15.5)	10 (22.2)	2 (18.2)	12 (12.5)	1 (11.1)	.467
Cerebral	40 (24.8)	13 (28.9)	5 (45.5)	21 (21.9)	1 (11.1)	.249
Spinal	2 (1.2)	0 (0.0)	0 (0.0)	2 (2.1)	0 (0.0)	1.000
Renal	25 (15.5)	6 (13.3)	3 (27.3)	15 (15.6)	1 (11.1)	.692
Visceral	33 (20.5)	9 (20.0)	2 (18.2)	21 (21.9)	1 (11.1)	.968
Peripheral	43 (26.7)	10 (22.2)	5 (45.5)	28 (29.2)	0 (0.0)	.100

Bold type indicates statistical significance. *AAD*, Acute aortic dissection; *BMI*, body mass index; *BSA*, body surface area; *COPD*, chronic obstructive pulmonary disease; *CPR*, cardiopulmonary resuscitation.

**Table E2:** Operative details, AAD subgroup

Parameter	Total AAD (N = 161)	Zone 0 (N = 45)	Zone 1 (N = 11)	Zone 2 (N = 96)	Zone 3 (N = 9)	*P* value
FET vascular graft diameter, mm, mean ± SD	27.8 ± 2.8	28.3 ± 1.5	28.7 ± 1.3	27.5 ± 3.0	27.7 ± 5.7	.110
FET stent graft diameter, mm, mean ± SD	28.0 ± 3.0	28.5 ± 2.8	29.3 ± 2.5	27.7 ± 2.8	28.1 ± 5.7	.121
FET stent graft length, mm, mean ± SD	135.0 ± 21.1	145.6 ± 29.6	153.2 ± 31.0	129.1 ± 9.5	123.3 ± 13.2	.138
FET device, n (%)						**<.001**
E-vita open neo	48 (29.8)	40 (88.9)	7 (63.6)	1 (1.0)	0 (0.0)	
E-vita open	103 (64.0)	4 (8.9)	3 (27.3)	89 (92.7)	7 (77.8)	
Thoraflex	10 (6.2)	1 (2.2)	1 (9.1)	6 (6.3)	2 (22.2)	
FET configuration, n (%)						**<.001**
Straight	125 (77.6)	25 (55.6)	4 (36.4)	89 (92.7)	7 (77.8)	
Trifurcated	26 (16.1)	19 (42.2)	6 (54.5)	1 (1.0)	0 (0.0)	
Branched	10 (6.2)	1 (2.2)	1 (9.1)	6 (6.3)	2 (22.2)	
Operative details, mean ± SD						
CPB time, min	265.1 ± 60.8	264.2 ± 54.5	297.9 ± 58.4	261.9 ± 63.4	262.7 ± 62.1	.186
Cross-clamp time, min	152.9 ± 43.7	164.6 ± 38.8	189.5 ± 50.9	143.4 ± 42.4	150.6 ± 40.9	**.001**
Distal ischemia time, min	29.9 ± 14.3	23.9 ± 12.4	43.9 ± 21.1	30.4 ± 12.1	36.8 ± 20.4	**<.001**
SCP, min	58.6 ± 21.2	57.2 ± 21.6	77.5 ± 30.5	55.5 ± 17.1	75.4 ± 29.7	**.028**
Lowest temperature, °C	24.6 ± 2.4	25.6 ± 1.2	25.6 ± 1.2	24.1 ± 2.5	22.4 ± 3.9	**<.001**
Transfusions and coagulation factors, mean ± SD						
pRBC, 250 mL units	4.28 ± 3.48	3.69 ± 2.81	4.82 ± 3.16	4.33 ± 3.75	6.00 ± 3.67	.216
Platelets, 250 mL units	2.08 ± 1.15	1.89 ± 1.15	2.36 ± 0.81	2.11 ± 1.20	2.33 ± 0.71	.385
FFP, 250 mL units	3.12 ± 3.13	3.73 ± 3.33	3.82 ± 2.04	2.95 ± 3.17	1.00 ± 1.58	**.024**
Fibrinogen, g	4.78 ± 2.32	4.76 ± 2.46	5.27 ± 1.35	4.76 ± 2.42	4.44 ± 1.59	.643
PCC, IU	2880 ± 1751	2793 ± 1751	3109 ± 1560	2795 ± 1835	3133 ± 1072	.657
Arterial cannulation site, n (%)						.062
Subclavian/axillary	125 (77.6)	35 (77.8)	7 (63.6)	78 (81.3)	5 (55.6)	
DTLC	28 (17.4)	8 (17.8)	2 (18.2)	16 (16.7)	2 (22.2)	
Others	8 (5.0)	2 (4.4)	2 (18.2)	2 (2.1)	2 (22.2)	
Aortic valve intervention, n (%)						**.004**
Replacement	25 (15.5)	10 (22.2)	2 (18.2)	13 (13.5)	0 (0.0)	
Reconstruction	95 (59.0)	19 (42.2)	3 (27.3)	67 (69.8)	6 (66.7)	
Aortic root intervention, n (%)						**.001**
Bentall	20 (12.4)	7 (15.6)	2 (18.2)	11 (11.5)	0 (0.0)	
David	6 (3.7)	4 (8.9)	0 (0.0)	2 (2.1)	0 (0.0)	
Isolated sinus replacement	19 (11.8)	13 (28.9)	0 (0.0)	6 (6.3)	0 (0.0)	
Concomitant TEVAR, n (%)	9 (5.6)	2 (4.4)	1 (9.1)	6 (6.3)	0 (0.0)	.844

Bold type indicates statistical significance. *AAD*, Acute aortic dissection; *FET*, frozen elephant trunk; *CPB*, cardiopulmonary bypass; *SCP*, selective cerebral perfusion; *pRBC*, packed red blood cells; *FFP*, fresh frozen plasma; *PCC*, prothrombin complex concentrate; *DTLC*, direct true lumen cannulation; *TEVAR*, thoracic endovascular aortic repair.

**Table E3:** Outcomes in the AAD subgroup

Parameter	Total AAD (N = 161)	Zone 0 (N = 45)	Zone 1 (N = 11)	Zone 2 (N = 96)	Zone 3 (N = 9)	*P* value
Postoperative ventilation, h, median (IQR)	25.0 (15.0-79.0)	25.0 (14.0-105.0)	92.0 (16.0-173.0)	24.0 (14.5-63.0)	34.5 (22.3-87.8)	.609
ICU stay, d, mean ± SD	6.7 ± 5.8	6.8 ± 6.1	8.4 ± 8.2	6.6 ± 5.5	4.7 ± 3.4	.690
Hospital stay, d, mean ± SD	16.3 ± 10.2	13.8 ± 9.7	18.4 ± 12.7	17.1 ± 9.8	17.6 ± 13.4	.151
Rethoracotomy, n (%)	20 (12.4)	5 (11.1)	2 (18.2)	10 (10.4)	3 (33.3)	.184
In-hospital mortality, n (%)	23 (14.3)	7 (15.6)	2 (18.2)	11 (11.5)	3 (33.3)	.249
Tracheotomy, n (%)	18 (11.2)	4 (8.9)	3 (27.3)	9 (9.4)	2 (22.2)	.148
Distal reintervention (TEVAR), n (%)	19 (11.8)	7 (15.6)	1 (9.1)	10 (10.4)	1 (11.1)	.840
Dialysis, n (%)	35 (21.7)	7 (15.6)	4 (36.4)	19 (19.8)	5 (55.6)	**.036**
New neurologic deficit, n (%)	13 (8.1)	4 (8.9)	3 (27.3)	6 (6.3)	0 (0.0)	.113
Stroke, n (%)∗	12 (92.3)	3 (75.0)	3 (100.0)	6 (100.0)	0 (0.0)	.538
Spinal cord injury, n (%)∗	2 (15.4)	0 (0.0)	1 (33.3)	1 (16.7)	0 (0.0)	.692
Long-term survival, % (95% CI)						.468
1 y	81.1 (73.9-86.5)	81.5 (66.3-90.3)	65.5 (23.6-88.3)	83.7 (74.4-89.9)	66.7 (28.2-87.8)	
2 y	78.2 (70.4-84.2)	73.3 (54.8-85.2)	65.5 (23.6-88.3)	82.3 (72.6-88.8)	66.7 (28.2-87.8)	
3 y	78.2 (70.4-84.2)	73.3 (54.8-85.2)	65.5 (23.6-88.3)	82.3 (72.6-88.8)	66.7 (28.2-87.8)	
4 y	76.6 (68.3-83.1)	N/A	N/A	80.5 (70.3-87.6)	66.7 (28.2-87.8)	
5 y	76.6 (68.3-83.1)	N/A	N/A	80.5 (70.3-87.6)	66.7 (28.2-87.8)	
6 y	72.1 (59.4-81.5)	N/A	N/A	75.5 (60.4-85.5)	N/A	

Bold type indicates statistical significance. *AAD*, Acute aortic dissection; *IQR*, interquartile range; *ICU*, intensive care unit; *TEVAR*, thoracic endovascular aortic repair; *N/A*, not applicable.

∗ Only including patients with new neurologic deficits (n = 13).

**Table E4:** Zone 0 versus zone 2

Parameter	Total (N = 214)	Zone 0 (N = 69)	Zone 2 (N = 145)	*P* value
Disease, n (%)				.069
AAD	141 (65.9)	45 (65.2)	96 (66.2)	
CAD	51 (23.8)	21 (30.4)	30 (20.7)	
TAA	22 (10.3)	3 (4.3)	19 (13.1)	
Age, y, mean ± SD	61.8 ± 10.6	61.6 ± 11.1	61.8 ± 10.3	.890
Sex, n (%)				.666
Male	153 (71.5)	48 (69.6)	105 (72.4)	
Female	61 (28.5)	21 (30.4)	40 (27.6)	
FET configuration, n (%)				**<.001**
Straight	164 (76.6)	30 (43.5)	134 (92.4)	
Trifurcated	42 (19.6)	38 (55.1)	4 (2.8)	
Branched	8 (3.7)	1 (1.4)	7 (4.8)	
Operative details, mean ± SD				
CPB time, min	261.3 ± 60.5	257.1 ± 54.0	263.2 ± 63.4	.493
Cross-clamp time, min	142.2 ± 45.6	153.5 ± 49.1	136.9 ± 43.0	**.013**
Distal ischemia time, min	28.5 ± 11.3	24.5 ± 10.9	30.4 ± 11.1	**<.001**
SCP time, min	56.0 ± 19.0	57.4 ± 19.9	55.4 ± 18.7	.479
Lowest temperature, °C	24.5 ± 2.3	25.6 ± 1.1	23.9 ± 2.5	**<.001**
Aortic valve intervention, n (%)				**.009**
Replacement	36 (16.8)	16 (23.2)	20 (13.8)	
Reconstruction	97 (45.3)	21 (30.4)	76 (52.4)	
Aortic root intervention, n (%)				**<.001**
Bentall	27 (12.6)	12 (17.4)	15 (10.3)	
David	10 (4.7)	6 (8.7)	4 (2.8)	
Isolated sinus replacement	20 (9.3)	13 (18.8)	7 (4.8)	
Postoperative course, mean ± SD				
ICU stay, d	6.5 ± 6.0	6.5 ± 6.1	6.6 ± 6.0	.885
Hospital stay, d	16.2 ± 9.7	15.0 ± 10.5	16.7 ± 9.3	.242
Rethoracotomy, n (%)	18 (8.4)	5 (7.2)	13 (9.0)	.672
In-hospital mortality, n (%)	19 (8.9)	7 (10.1)	12 (8.3)	.653
Dialysis, n (%)	38 (17.8)	9 (13.0)	29 (20.0)	.213
Long-term survival, %, median (95% CI)				.935
1 y	84.3 (78.5-88.7)	86.5 (75.6-92.7)	83.4 (76.0-88.6)	
2 y	80.5 (74.1-85.5)	79.4 (65.9-88.0)	80.8 (73.0-86.5)	
3 y	80.5 (74.1-85.5)	79.4 (65.9-88.0)	80.8 (73.0-86.5)	
4 y	79.5 (72.8-84.7)	79.4 (65.9-88.0)	79.7 (71.6-85.7)	
5 y	76.5 (68.6-82.7)	N/A	76.7 (67.7-83.5)	
6 y	73.8 (64.0-81.2)	N/A	73.9 (63.4-81.9)	

Bold type indicates statistical significance. *AAD*, Acute aortic dissection; *CAD*, chronic aortic dissection; *TAA*, thoracic aortic aneurysm; *FET*, frozen elephant trunk; *CPB*, cardiopulmonary bypass; *SCP*, selective cerebral perfusion; *ICU*, intensive care unit; *N/A*, not applicable.
